# White matter microstructure and gray matter density in 12-year-old preterm born children

**DOI:** 10.1038/s41390-025-04451-w

**Published:** 2025-10-06

**Authors:** Annette Karimi, Ylva Fredriksson Kaul, Olga Kochukhova, Martin Johansson, Cecilia Montgomery, Markus Fahlström, Sven Haller, Lena Hellström-Westas, Johan Wikström

**Affiliations:** 1 Department of Surgical Sciences, Neuroradiology, Uppsala University, Uppsala, Sweden; 2 Radiology Department, Uppsala University Hospital, Uppsala, Sweden; 3 Departments of Women’s and Children’s Health, Uppsala University, Uppsala, Sweden; 4 Departments of Psychology, Uppsala University, Uppsala, Sweden; 5 Department of Surgical Sciences, Molecular Imaging and Medical Physics, Uppsala University, Uppsala, Sweden; 6 Division of Radiodiagnostic and Interventional Radiology, Faculty of Medicine, University of Geneva, Geneva, Switzerland

## Abstract

**Background:**

To investigate white matter microstructure and gray matter densities in 12-year-old children born very preterm and evaluate potential effects of antenatal steroids and intraventricular hemorrhage (IVH).

**Methods:**

Brain MRI (3Tesla) was performed in 57 children born very preterm, 16 with IVH and 41 treated with antenatal corticosteroids, and in 22 full-term controls. White matter microstructure and gray matter density were compared between groups using tract-based spatial statistics (TBSS) and voxel-based morphometry (VBM).

**Results:**

Preterm-born children showed no differences in white matter fractional anisotropy (FA) compared to controls, but showed increased or decreased gray matter density in several regions. In the preterm group, lower gestational age was associated with reduced FA in the anterior commissure and the anterior parts of the inferior fronto-occipital fasciculus. Antenatal steroid exposure did not affect FA or gray matter density. IVH was associated with decreased gray matter density in the temporo-occipital cortex.

**Conclusion:**

Very premature birth is associated with alterations in gray matter density, and there is a correlation between gestational age and white matter integrity. Antenatal steroid exposure does not affect white matter integrity or gray matter density, but IVH exposure is associated with locally decreased gray matter density.

**Impact:**

- Prematurely born adolescents show differences in gray matter density in several regions compared to controls, with both increased and decreased values observed.
- In prematurely born adolescents, there is a correlation between lower gestational age and reduced white matter integrity.
- Assessment of white matter microstructure using tract-based spatial statistics (TBSS) and gray matter volumes using voxel-based morphometry (VBM) in the same cohort of subjects shows alterations in regions involving the same neural circuit.

## Introduction

Very preterm birth increases risk for perinatal brain injury.^1^ During the last decade, around 1% of all children in Sweden were born very preterm.^2^ With improvements in neonatal care the survival rates for preterm-born children have risen in recent decades, but there is still an increased risk of perinatal cerebral damage. Due to immature cerebral autoregulation and ongoing neurodevelopment with neuron production, migration and maturation the neonatal brain is especially vulnerable to complications such as infection, inflammation, hemorrhage and hypoxia-ischemia.^3–6^ The introduction of antenatal steroid therapy has reduced neonatal mortality and decreased the risk for perinatal complications including respiratory stress, intraventricular hemorrhage (IVH) and necrotizing enterocolitis.^7^ However, the use of antenatal steroids has shown conflicting findings in research. In very preterm-born infants, the antenatal administration of steroids has been shown to be a neuroprotective factor while the absence of treatment is associated with adverse outcomes.^8^ However, for children born at term and even children born late preterm (34–36 weeks of gestational age) the treatment is linked to poorer neurodevelopment.^9,10^ Further, a meta-analysis by Ninan et al. showed that 40% of children treated with early antenatal steroids were born at term, making the exposure an unnecessary risk.^10^ Previous investigations with brain MRI in children born preterm have shown macro-structural differences compared to full-term controls, including thinning of the corpus callosum, ventricle dilatation, diffuse white matter lesions and reduced white matter volumes.^11–13^ These assessments were based on qualitative and semi-quantitative measurements and showed clear associations with neurodevelopmental impairments affecting vision, language and executive functions.^14–17^ In a previous study, we found structural brain MRI abnormalities and ventricular dilatation, indicating white matter reduction in 12-year-old children born very preterm and a correlation with visual function.^18^ Together, these findings confirm that structural brain damage among children born preterm persists into adolescence and is associated with neurodevelopmental impairments.

To further investigate abnormalities in white matter microstructure and changes in gray and white matter volumes, advanced MRI techniques such as diffusion tensor imaging (DTI) and voxel-based morphometry (VBM) can provide further insight.^19–21^

DTI has been used to characterize the microstructure of white matter tracts. Fractional anisotropy (FA) measures the degree to which diffusion is anisotropic, and a reduction of FA is interpreted as a sign of reduced white matter integrity. Nagy et al. observed reduced FA in the posterior corpus callosum, fornix and internal capsule in preterm-born children and adolescents aged 11–18 years.^22,23^ Eikenes et al. showed significantly reduced FA bilaterally in several major white matter tracts, especially in the posterior region, among young adults with very low birth weight compared to controls.^24^ This is in line with our and other previous studies, suggesting that the posterior regions are particularly vulnerable to white matter injury in children born very preterm.^17,18,25^

In a few previous studies using VBM, changes in gray matter volume were observed among adolescents and even among adults born very preterm.^26–28^ One consistent finding is the volume reduction of the temporal lobe. Regional volume changes, both decreases and increases, were also observed in other regions, including the thalamus, caudate nucleus and hippocampus. A recently published study showed that 10-year-old children born extremely preterm had several regions with altered gray and white matter volumes, and also that neonatal low-grade IVH (I-II) was associated with decreased volumes.^27^ Previous studies have thus shown microstructural differences between prematurely born adolescents and controls in different regions. By assessing white matter microstructure and gray matter volumes in the same cohort of subjects, the current study might add information to existing knowledge, especially if changes were identified in regions involving the same neural circuit with different methods. It is of further interest to assess if previous findings of microstructural alterations persist also in a more recent cohort, bearing in mind advancements in neonatal care.

The aim of the present study was to investigate differences in brain microstructure between 12-year-old children born very preterm and age-matched controls. Using advanced MRI methods for quantitative analysis, including tract-based spatial statistics (TBSS) and VBM, we evaluated regional white matter microstructure and gray matter densities, respectively. This whole-brain approach allows for a comprehensive assessment of structural differences across the brain. We also investigated potential influence of gestational age, antenatal steroids, and IVH on brain structure in the preterm group. We hypothesized that the preterm group would have reduced FA and alterations in gray matter densities compared to the full-term controls.

## Material and methods

### Participants

**Fig. 1:**
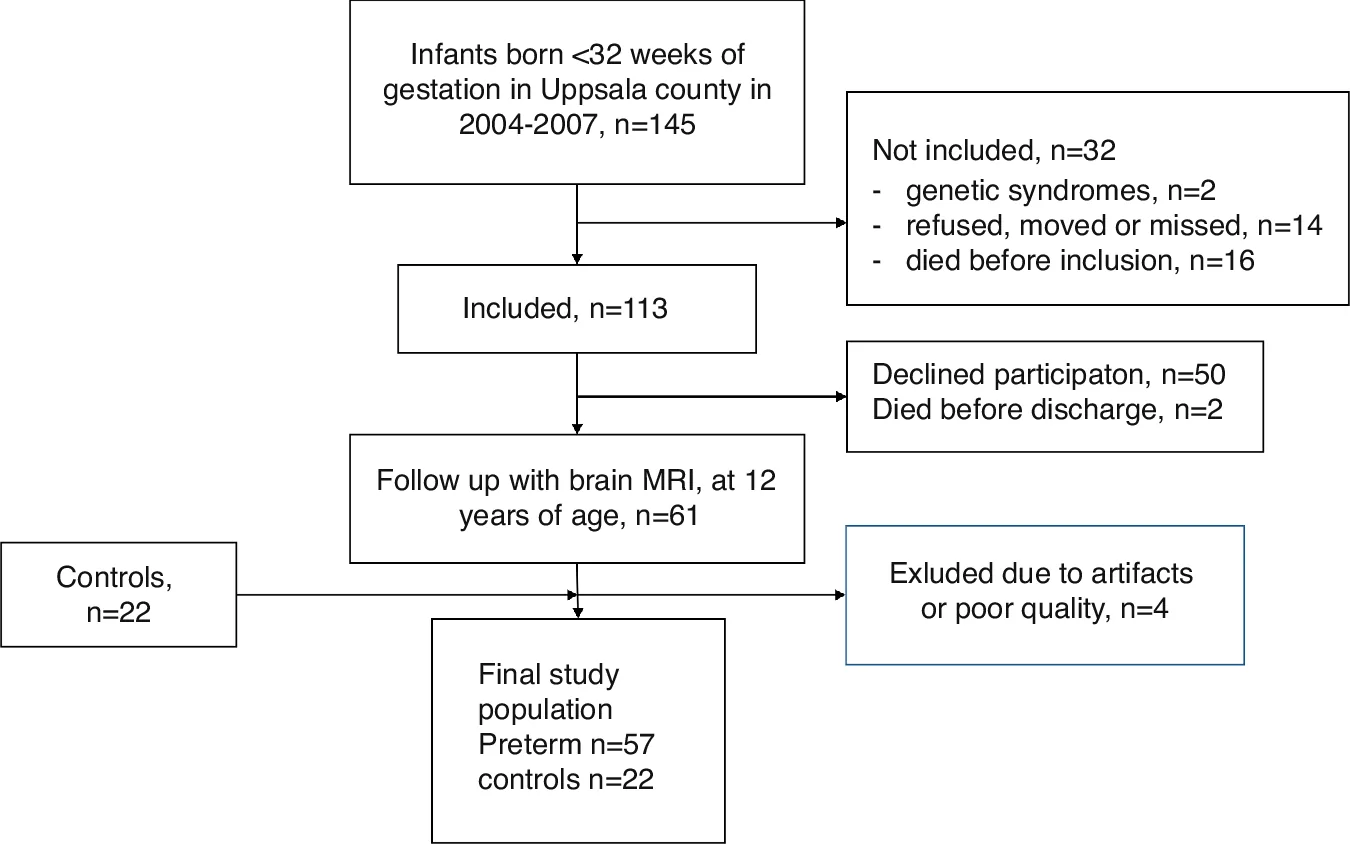
Flowchart describing the study population.

This study is part of the LOngitudinal multidisciplinary study of VISuomotor capacity in very preterm infants (LOVIS), a population-based prospective follow-up study of 113 very preterm infants born at <32 weeks of gestational age in 2004–2007 in Uppsala County, Sweden.^29^ Fifty-seven 12-year-old children born very preterm, and 22 full term controls (born >37 weeks of gestational age, in 2005–2009) were included. The MRI assessments were performed at a mean age of 12.8 years (median 12.6 years, range 11.7 to 14.6) among the preterm children, compared to the controls mean 13.0 (median 13.0 years, range 12.2 to 13.8), respectively. A flow-chart describing the very preterm study population is shown in Fig. 1. Of the included 113 subjects two died before discharge and 50 declined their participation. After the MRI exams, 4 subjects were excluded due to artifacts or poor exam quality. Parental written consent was obtained after written and oral information to the child and the parents. The LOVIS study had ethical approval from the Regional Research Ethics Committee in Uppsala (Application number 03/665 and 2016/400).

### Statistical analysis of perinatal characteristics

Normally distributed and skewed background characteristics were compared using the independent samples T-Test or Mann–Whitney U-Test, as appropriate. For categorical background characteristics, Pearson’s Chi-Square Test or Fisher’s Exact Test were used. A *p*-value < 0.05 was considered statistically significant. The statistical analysis was performed using SPSS version 28.0 (IBM SPSS Statistics for Windows, IBM Cooperation, Armonk, NY).

### Brain MR imaging

Brain MRI examinations took place between 2018 and 2022 at the Radiology Department at Uppsala University Hospital. MRIs were performed without sedation using a 3.0 Tesla MR system (dSTREAM, Achieva, Philips Healthcare, Best, the Netherlands). The protocol included an axial three-dimensional T1 weighted sequence with TR/TE/flip angle: 8 ms/ 4 ms/8°, acquired resolution 1 × 1 × 1 mm (slice thickness 1 mm), and a DTI sequence with TR/TE/flip angle: 3739 ms/86 ms/90°, acquired resolution 1.75 × 1.75 × 2 mm (slice thickness 2 mm), *b* values of 0 and 800 s/mm^2^ (*n* = 62) or 0 and 1000 s/mm^2^ (*n* = 17), and 48 diffusion directions.

Further sequences were acquired and analysed to exclude brain pathologies: an axial T2 weighted sequence with TR/TE/flip angle: 3381 ms/80 ms/90°, acquired resolution 0.45 × 0.45 ×4 (4 mm slice thickness) and a sagittal 3D Flair sequence with TR/TE/TI/flip angle: 4800 ms/308 ms/1650 ms/90°, acquired resolution 1.11 × 1.11 × 1.12 mm (slice thickness 1.12 mm).

### White matter analysis of DTI data

For the TBSS analysis of the DTI data, the FSL software package (https://fsl.fmrib.ox.ac.uk/fsl) was used. FA maps were created for each subject based on the DTI data. During the standardized pre-processing eddy-current and motion correction was performed. Brain extraction was applied using BET (brain extraction tool, part of FSL) and tissue-type segmentation. Using non-linear registration, all subjects’ FA images were aligned to a 1 × 1 × 1 mm standard space resulting in a mean FA tract skeleton. A threshold ≥0.2 for the FA was used to only include major white matter tracts. Voxel-wise statistical analysis was done using a non-parametric permutation testing in the FSL “randomise” function using a threshold free cluster enhancement multiple comparisons correction. Corrected *p*-values < 0.05 are considered significant. First, using all participants, a group comparison was performed for patients versus controls. Second, considering only the patients, two separate group analyses were performed for present/absent steroid treatment and present/absent IVH. Additionally, only in the patient group, a correlation analysis of GA was performed. The relevant anatomical regions were localised and named in accordance with John Hopkins University DTI-based white matter atlases.^30^

### Gray matter VBM analysis of T1 Data

Also, the VBM analyses were performed using the FSL software package https://fsl.fmrib.ox.ac.uk/fsl. Similar to the TBSS analyses, an automated image analysis was performed including the standard processing steps: Brain extraction (BET, part of FSL), tissue type segmentation (part of FSL) and nonlinear transformation into a MNI (MNI152, Montreal Neurological Institute) reference space. By aligning all images in a standardized MNI space, a correction for brain volume is also performed. Voxel-wise statistical analyses was again performed in FSL “randomise” for the same comparisons described in the TBSS analysis above. The significant regions were identified with the Harvard–Oxford cortical and subcortical atlases and clusters specified according to coordinates with maximum values (Table 2).^31,32^

## Results

Perinatal characteristics of the included 57 children (26 girls) born very preterm and those who did not participate (*n* = 56) are shown in Table 1. The non-participants had a higher proportion of Cesarean section compared to the study children (69.6% versus 50.9%, *p* = 0.04). No other significant differences regarding perinatal characteristics between the two groups were observed. Of the participating preterm born children, 13 had IVH grade 1, one had IVH grade 2, two had IVH grade 3 and four children had PVL as diagnosed on neonatal ultrasound. For comparison, 22 full-term age-matched controls (11 girls) were included.

**Table 1:** Perinatal characteristics

Perinatal characteristics	Children who performed MRI assessment (*n* = 57)	Children not participating (*n* = 56)	*p*-value
Gestational age, median (min, max), w	29.4 (22.7,31.9)	29.1 (22.3,31,9)	0.9
Birth weight, mean (SD) g	1224.6 (46.7)	1170.5 (47.3)	0.4
Small for gestational age, *n* (%)	10 (17.5)	12 (21.4)	0.6
Female, *n* (%)	26 (45.6)	25 (44.6)	0.9
Multiple birth, *n* (%)	21 (36.8)	14 (25)	0.2
Antenatal corticosteroids, *n* (%)	41 (71.9)	37 (66.1)	0.5
Cesarean section, *n* (%)	29 (50.9)	39 (69.6)	0.04
PVL, *n* (%)	4 (7)	3 (5.4)	1.0
IVH, *n* (%)	16 (28.1)	8 (14.3)	0.07
IVH grade 1, *n* (%)	13 (22.8)	6 (10.7)	
IVH grade 2, *n* (%)	1 (1.8)	0	
IVH grade 3, *n* (%)	2 (3.5)	1 (1.8)	
IVH grade 4, *n* (%)	0	1 (1.8)	
Severe brain injury (PVL or IVH grade 3), *n* (%)	5 (8.8)	4 (7.1)	1.0
ROP – Any, *n*(%)	14 (24.6)	18 (32.1)	0.4
ROP ≥ 3, *n* (%)	2 (3.6)	7 (12.5)	0.09
ROP, treated, *n* (%)	1 (1.8)	5 (8.9)	0.1
Sepsis, *n* (%)	13 (22.8)	9 (16.1)	0.4
PDA, *n* (%)	12 (21.1)	13 (23.2)	0.8
Operated PDA, *n* (%)	8 (14.0)	7 (12.5)	0.2
BPD, *n* (%)	11 (19.3)	14 (25)	0.5
NEC, *n* (%)	0 (0)	0 (0)	

### White matter analysis of DTI data

**Fig. 2:**
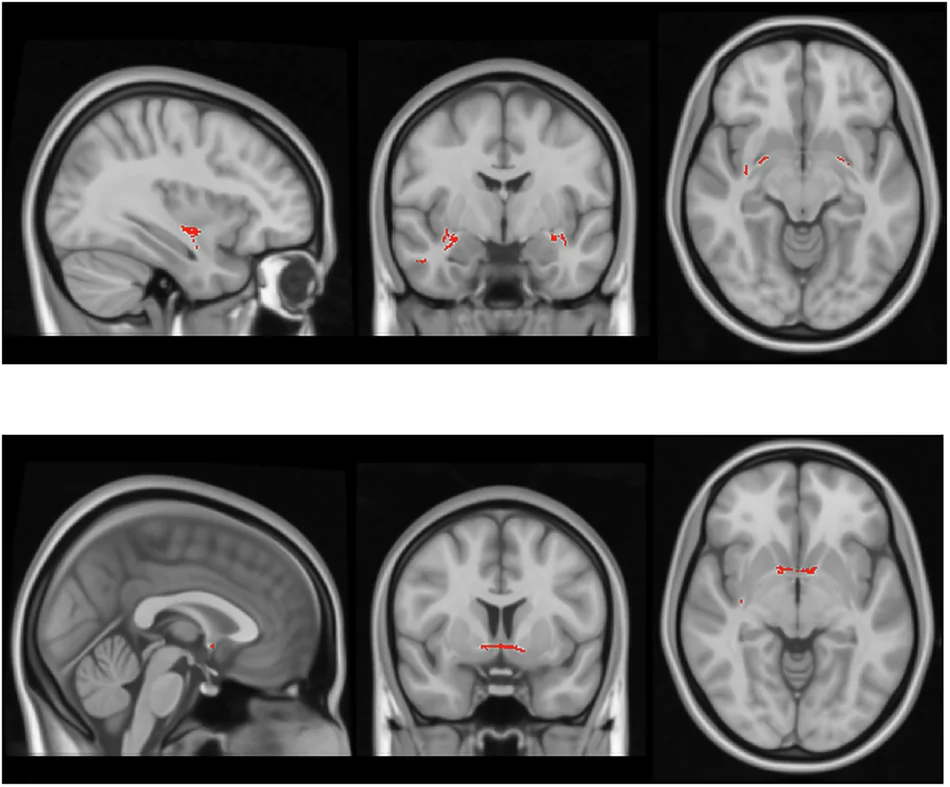
TBSS correlation analysis among preterm born adolescents showing association between lower gestational age and decreased FA values in the anterior commissure extending to the anterior parts of the inferior fronto-occipital fasciculus (red).

There were no differences in FA between the preterm group and controls. Among the preterm children, lower gestational age was associated with significantly reduced FA values in the anterior commissure extending to the anterior parts of the inferior fronto-occipital fasciculus (Fig. 2). No significant differences in FA were found in relation to treatment with antenatal steroids in the preterm group. Further, there were no significant differences in FA among preterm-born with or without IVH.

### Gray matter VBM analysis of T1 data

Compared to the full-term controls, the preterm group had decreased gray matter densities in the thalamus bilaterally, the left caudate and bilaterally in the hippocampus as well as in the posterior division of the right middle temporal gyrus, parts of the lingual gyrus and the left posterior part of the cerebellum.

**Fig. 3:**
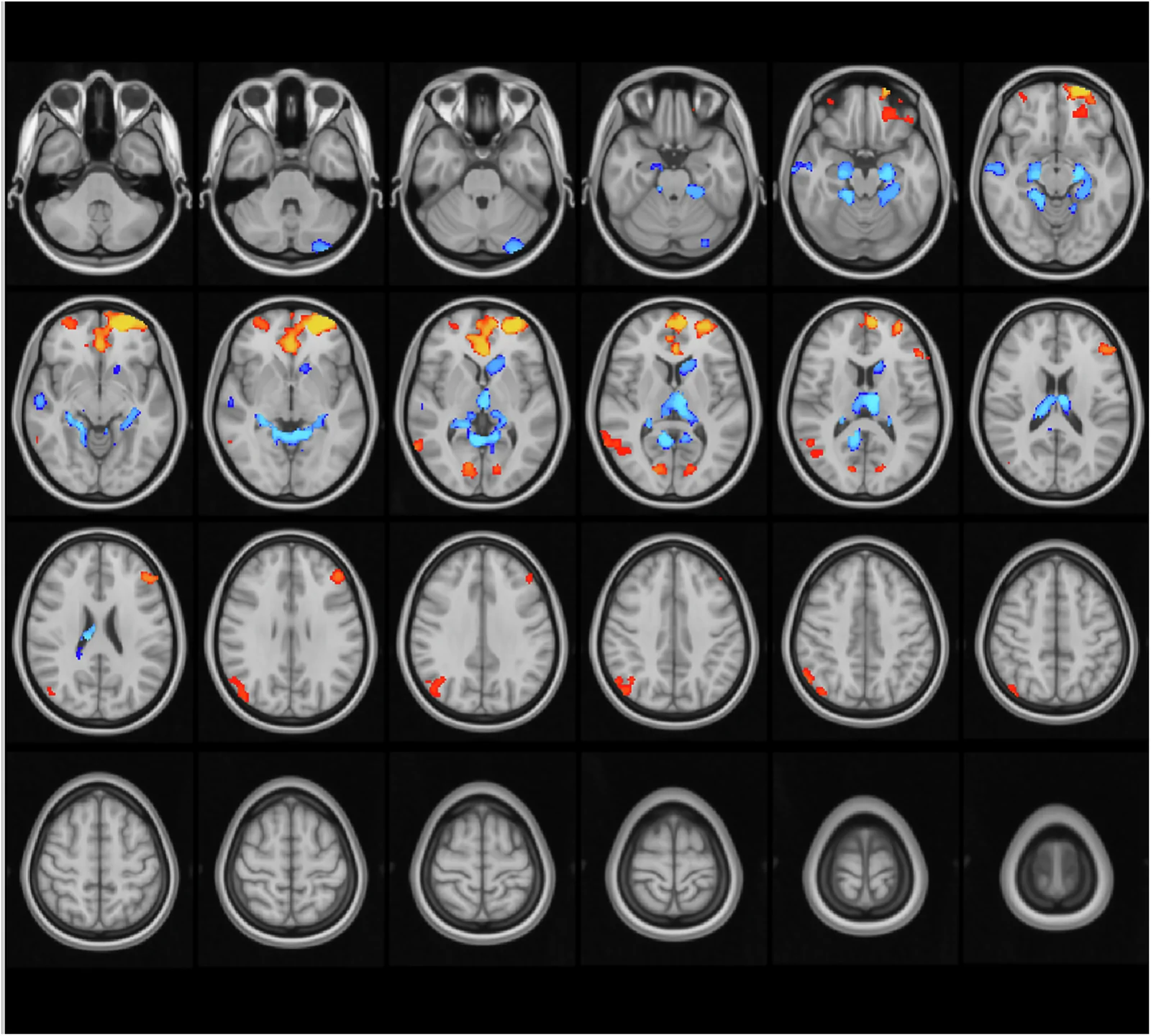
Voxel-based morphometry (VBM) group comparison showing regions with decreased gray matter densities (blue) and increased gray matter densities (red) among preterm-born adolescents compared to controls.

Increased gray matter densities were found bilaterally in the frontal cortices, in the right temporo-occipital part of the middle temporal gyrus, the right occipital cortex and bilaterally in the intracalcarine cortex and the anterior part of the cingulate gyrus. The most prominent clusters of altered gray matter densities showed a pattern of bilaterality (Fig. 3 and Table 2).

**Table 2:** Regions with differences in gray matter density between preterm-born adolescents (*n* = 57) and term-born controls (*n* = 22), analysed with voxel-based morphometry (VBM).

Anatomical region	Hemisphere	Coordinates in MNI152		
		Max x	Max y	Max z
Preterm < Controls				
Caudate nucleus	left	51	72	38
Thalamus Hippocampus Lingual gyrus	bilaterally	48	55	42
Middle temporal gyrus (posterior part)	right	16	56	30
Posterior part cerebellum	left	59	19	20
Preterm > Controls				
Frontal cortex Cingulate gyrus (anterior part)	left	58	94	30
	right	31	91	33
Middle frontal gyrus	left	67	80	46
Middle temporal gyrus (temporo-occipital part)	right	13	33	38
Intracalcarine cortex	right	38	24	37
	left	52	23	38
Lateral occipital cortex	right	18	31	57

In the premature group, there was no correlation between gestational age and gray matter density. No difference in gray matter densities was observed among preterm-born children in relation to antenatal steroids. The preterm-born children with neonatal IVH showed decreased gray matter in a small region in the temporo-occipital cortex, as compared to those without IVH.

## Discussion

The present study evaluated white matter microstructure and gray matter density with TBSS and VBM in 12-year-old children born very preterm and controls.

We found no group differences between the preterm children and controls as regards white matter tracts analysed with TBSS. In contrast, Nagy et al. found decreased FA in white matter tracts in very preterm children, compared to full-term controls, in the posterior corpus callosum, fornix and external capsula.^23^ Alterations of white matter tracts were also reported by Vangberg et al., in a study showing decreased FA in the posterior part of the capsula interna, centrum semiovale and occipital periventricular white matter in very-low-birthweight adolescents.^33^ A recent meta-analysis demonstrated that the most robust WM alterations in individuals born very preterm were found in the corpus callosum, the bilateral thalamus and the left superior longitudinal fasciculus, and were present from infancy to young adulthood.^21^

A possible explanation for differences between our study and previous reports may be the time of birth. The children in our cohort were born in 2004–2007, while the cohorts in Vangberg et al. and Nagy et al. were born in 1986–1988 and 1988–1993, respectively. Improvements in neonatal care in the time period between the studies could explain the absence of group differences in our study.

As shown in Fig. 2, the present study demonstrated a significant correlation between low gestational age and reduced FA values in the anterior commissure and partly involving the inferior fronto-occipital fasciculus among the children born very preterm. Also, Nagy at al. found a correlation between gestational age and reduced FA, albeit involving other regions such as external capsule, corpus callosum and the fornix.^23^

A correlation between lower gestational age and reduced FA among very preterm born children suggests a higher risk of white matter injury among children born extremely preterm (<28 weeks of gestational age) compared to preterm born of higher gestational age. Therefore, it might be of interest to separately analyse adolescents born extremely preterm, as in a previous study by Kvanta et al. comparing adolescents born before 27 weeks of gestation with term-born controls. The extremely preterm-born children showed significant regional volumetric differences with increases and decreases in both gray and white matter compared to controls.^27^ Among the very preterm born children in our study only 19% were born extremely preterm, mean gestational age was 29.4 weeks (range 22.7 to 31.9 weeks). Thus, we did not have the power to perform a subgroup analysis of the extremely preterm children.

Assessment with VBM demonstrated differences in gray matter volumes between the preterm-born children and the group of term-born controls. Clusters of decreased gray matter among the preterm-born children were found in subcortical gray matter nuclei, such as the left caudate nucleus and bilaterally in the thalamus as well as in the lingual gyrus extending to the hippocampus. Further, the posterior part of the middle temporal gyrus showed decreased gray matter volume. Increased gray matter among preterm-born children was on the other hand found bilaterally in the frontal cortex and the anterior part of the cingulate gyrus.

Also, previous studies have shown alterations in gray matter volumes between preterm-born children and children born at term.^23,27,28^ Kvanta et al. found the most prominent cluster of gray matter reduction in the temporal lobes and the most prominent cluster of gray matter increase in the cingulate gyrus, regions overlapping our findings. Also, Nosarti et al. found regions with decreased gray matter volumes in the temporal lobe, but also extending medially, involving subcortical gray matter such as the thalamus and the caudate. Increased volumes were found in the frontal cortex and middle temporal gyri. Similarly, Nagy et al. found decreased gray matter bilaterally in the temporal lobes, the caudate nuclei, hippocampi and thalami. In our study, some clusters were unilateral, but the main clusters showed a pattern of bilaterality.

In line with our hypothesis, we could identify alterations in functionally related white matter and gray matter regions with different microstructural techniques. Alterations were found in structures involving visual processing, such as the intracalcarine cortex and occipital parts of the middle temporal gyrus as well as parts of the inferior fronto-occipital fasciculus, analysed with VBM and TBSS, respectively. These findings are in agreement with our previous work that showed a correlation between adverse visual development and structural MRI findings in occipital regions among preterm-born children compared to controls.^18^ Similarly, Sripada at al. showed a positive correlation between visual perception and FA values in white matter tracts including the inferior fronto-occipital fasciculus.^34^ Furthermore, prior studies involving our cohort have identified functional impairments, including deficits in language, reading, motor abilities and cognitive functions as well as disruptions in emotional processing. These functional difficulties may be corresponding to structural alterations observed in gray matter regions.^9,35,36^

The current results demonstrate that disturbance of brain development due to preterm birth not only results in tissue loss, but in a pattern of regions of both decreased and increased gray matter density. Some of the regions with altered densities were found adjacent to each other, and some areas were located in spatially distant parts. In the temporal lobe, the posterior part of the middle temporal gyrus demonstrated decreased gray matter density while the temporo-occipital part demonstrated increased gray matter density among the very preterm-born children. Similarly, the lingual gyrus showed bilaterally decreased gray matter density adjacent to the intracalcarine cortex showing bilaterally increased gray matter density. Typical brain development is determined by a complex time series of critical events such as neuron production, migration and maturation, synaptogenesis and synaptic pruning.^37^ A possible interpretation of our findings is that in case of a focal injury, neural interconnections lead to abnormal development in primarily unaffected adjacent or distant regions. This corresponds to the hypothesis of a neuroplastic network, where developmental disturbance in a brain region results in a cascade of alterations in other regions.^26–28^ It is therefore not unexpected to find gray matter alterations, increases as well as decreases, in regions both nearby and distant from each other. Correspondingly, Hollund et al. reported associations between motor function and FA among children with very low birth weight in both positive and negative direction. A possible interpretation of a negative correlation between FA and motor function is the effect of general axon loss and poorer axonal branching in crossing fiber regions.^38^

Gray matter growth typically follows an “inverted U-shape”, representing the proliferation and organization of synapses, with a rapid synaptogenesis starting around the third trimester and continuing during the first years of life. This is followed by a reduced speed in gray matter growth, representing synaptic pruning, until reaching the density levels of adults with varying peak timing across different cortical regions.^39^ Instead, white matter growth follows a linear pattern representing the myelinization process continuing until the third decade of life.^37^

Therefore, regional altered gray matter densities among preterm-born adolescents might be a consequence of perinatal complications such as infection, inflammation, peri- or intraventricular hemorrhage and hypoxia-ischemia disturbing the fine-tuned processes taking place during the third trimester. Regions with increased gray matter densities among preterm-born adolescents compared to controls might represent delayed synaptic pruning or apoptosis.^28^ Likewise, white matter in preterm-born infants is particularly vulnerable to injury during the third trimester when crucial processes in myelination occur.^5^

A strength of the present study is its population-based study design and the follow-up beyond term equivalent age or early childhood to investigate persisting alterations during adolescence. Another strength is the use of two methods to analyse the same cohort from different perspectives and to assess different impacts of very preterm birth on brain structure. Further, we could reproduce parts of our results, such as alterations in regions involving visual processing, using different MRI techniques which strengthens our findings. However, the used methods also have limitations. A key limitation is the nonlinear registration used in both TBSS and VBM, which may introduce inaccuracies if brains vary significantly between the subjects. In TBSS, this may lead to misalignment of the white matter tracts, in VBM to inaccuracies in regions with high variability, like the cortex. Further, the mean FA skeleton in TBSS may reduce complex white matter anatomy to a simplified pathway and may miss changes in peripheral regions or crossing fiber regions. In VBM, the spatial smoothing, reducing spatial specificity, can obscure subtle differences in adjacent brain regions. Another limitation of this study is the limited population with small amounts of children in the stratified groups for perinatal risk factors. Thus, we could not perform a separate analysis for the group of extremely preterm-born children compared to controls. Another possible limitation of this study was its moderate follow-up rate. However, apart from Cesarean section, there were no differences between participating children and those who did not participate regarding perinatal characteristics. It is unlikely that the results of the evaluated cohort are caused by selection bias and we believe that our results are generalizable to other very preterm populations in high-income countries. Further, we are aware that the post-processing software we used is created for adult brains. To our knowledge, there is no special image post-processing software for adolescents and the software has been used in similar earlier studies.

## Conclusion

Alterations in gray matter microstructure in preterm-born children persist into adolescence. Further, lower gestational age is associated with reduced white matter integrity. Given the great increase in number of surviving very preterm born children our results emphasize the importance of limiting preventable risk factors and of optimizing conditions for brain growth and development during the neonatal period.

## Data Availability

The datasets generated and analysed during the current study are available from the corresponding author on reasonable request.

## References

[1] Group, E. et al. One-year survival of extremely preterm infants after active perinatal care in Sweden. *JAMA* **301**, 2225–2233 (2009). 19491184 10.1001/jama.2009.771

[2] Norman, M. et al. Association between year of birth and 1-year survival among extremely preterm infants in sweden during 2004-2007 and 2014-2016. *JAMA* **321**, 1188–1199 (2019). 30912837 10.1001/jama.2019.2021 PMC6439685

[3] de Vries, L. S., Benders, M. J. & Groenendaal, F. Progress in neonatal neurology with a focus on neuroimaging in the preterm infant. *Neuropediatrics* **46**, 234–241 (2015). 26121069 10.1055/s-0035-1554102

[4] Khwaja, O. & Volpe, J. J. Pathogenesis of cerebral white matter injury of prematurity. *Arch. Dis. Child Fetal Neonatal Ed.* **93**, F153–F161 (2008). 18296574 10.1136/adc.2006.108837 PMC2569152

[5] van Tilborg, E. et al. Origin and dynamics of oligodendrocytes in the developing brain: implications for perinatal white matter injury. *Glia* **66**, 221–238 (2018). 29134703 10.1002/glia.23256 PMC5765410

[6] Inder, T. E., Wells, S. J., Mogridge, N. B., Spencer, C. & Volpe, J. J. Defining the nature of the cerebral abnormalities in the premature infant: a qualitative magnetic resonance imaging study. *J. Pediatr.* **143**, 171–179 (2003). 12970628 10.1067/S0022-3476(03)00357-3

[7] Sotiriadis, A. et al. Neurodevelopmental outcome after a single course of antenatal steroids in children born preterm: a systematic review and meta-analysis. *Obstet. Gynecol.* **125**, 1385–1396 (2015). 26000510 10.1097/AOG.0000000000000748

[8] Axford, S. B. et al. Risk factor effects on neurodevelopment at 2 years in very preterm children: a systematic review. *Pediatrics* **155**, 1–27 (2025). 10.1542/peds.2024-069565 40368397

[9] Hedenius, M. et al. Predictors of language and reading outcomes in 12-year-old children born very preterm. *Acta Paediatr.* **114**, 100–108 (2024). 10.1111/apa.17408 PMC11627455 39222008

[10] Ninan, K. et al. The proportions of term or late preterm births after exposure to early antenatal corticosteroids, and outcomes: systematic review and meta-analysis of 1.6 million infants. *BMJ* **382**, e076035 (2023). 37532269 10.1136/bmj-2023-076035 PMC10394681

[11] Nosarti, C. et al. Adolescents who were born very preterm have decreased brain volumes. *Brain* **125**, 1616–1623 (2002). 12077010 10.1093/brain/awf157

[12] Griffiths, S. T. et al. Cerebral magnetic resonance imaging findings in children born extremely preterm, very preterm, and at term. *Pediatr. Neurol.* **49**, 113–118 (2013). 23859857 10.1016/j.pediatrneurol.2013.03.006

[13] Skranes, J. S. et al. Cerebral magnetic resonance imaging (MRI) of very low birth weight infants at one year of corrected age. *Pediatr. Radio.* **22**, 406–409 (1992). 10.1007/BF02013497 1437361

[14] Skranes, J. et al. Abnormal cerebral MRI findings and neuroimpairments in very low birth weight (VLBW) adolescents. *Eur. J. Paediatr. Neurol.* **12**, 273–283 (2008). 17933566 10.1016/j.ejpn.2007.08.008

[15] Nosarti, C. et al. Corpus callosum size and very preterm birth: relationship to neuropsychological outcome. *Brain* **127**, 2080–2089 (2004). 15289268 10.1093/brain/awh230

[16] Stewart, A. L. et al. Brain structure and neurocognitive and behavioural function in adolescents who were born very preterm. *Lancet* **353**, 1653–1657 (1999). 10335784 10.1016/s0140-6736(98)07130-x

[17] Aukland, S. M. et al. Ventricular dilatation in ex-prematures: only confined to the occipital region? MRI-based normative standards for 19-year-old ex-prematures without major handicaps. *Acta Radio.* **55**, 470–477 (2014). 10.1177/0284185113497476 23939381

[18] Karimi, A. et al. Brain MRI findings and their association with visual impairment in young adolescents born very preterm. *Neuroradiology* **66**, 145–154 (2024). 37870588 10.1007/s00234-023-03235-5 PMC10761469

[19] Feldman, H. M., Yeatman, J. D., Lee, E. S., Barde, L. H. & Gaman-Bean, S. Diffusion tensor imaging: a review for pediatric researchers and clinicians. *J. Dev. Behav. Pediatr.* **31**, 346–356 (2010). 20453582 10.1097/DBP.0b013e3181dcaa8b PMC4245082

[20] Zhou, L. et al. Brain gray and white matter abnormalities in preterm-born adolescents: a meta-analysis of voxel-based morphometry studies. *PLoS ONE* **13**, e0203498 (2018). 30303972 10.1371/journal.pone.0203498 PMC6179190

[21] Zhou, L. et al. Alteration of fractional anisotropy in preterm-born individuals: a systematic review and meta-analysis. *J. Obstet. Gynaecol.* **44**, 2371956 (2024). 38984803 10.1080/01443615.2024.2371956

[22] Nagy, Z. et al. Preterm children have disturbances of white matter at 11 years of age as shown by diffusion tensor imaging. *Pediatr. Res.* **54**, 672–679 (2003). 12904607 10.1203/01.PDR.0000084083.71422.16

[23] Nagy, Z. et al. Structural correlates of preterm birth in the adolescent brain. *Pediatrics* **124**, e964–e972 (2009). 19858152 10.1542/peds.2008-3801

[24] Eikenes, L., Lohaugen, G. C., Brubakk, A. M., Skranes, J. & Haberg, A. K. Young adults born preterm with very low birth weight demonstrate widespread white matter alterations on brain Dti. *Neuroimage* **54**, 1774–1785 (2011). 20965255 10.1016/j.neuroimage.2010.10.037

[25] Skranes, J. S. et al. Cerebral MRI findings in very-low-birth-weight and small-for-gestational-age children at 15 years of age. *Pediatr. Radio.* **35**, 758–765 (2005). 10.1007/s00247-005-1446-2 15864579

[26] Allin, M. et al. Effects of very low birthweight on brain structure in adulthood. *Dev. Med Child Neurol.* **46**, 46–53 (2004). 14974647 10.1017/s0012162204000088

[27] Kvanta, H. et al. Extreme prematurity and perinatal risk factors related to extremely preterm birth are associated with complex patterns of regional brain volume alterations at 10 years of age: a voxel-based morphometry study. *Front. Neurol.* **14**, 1148781 (2023). 37273719 10.3389/fneur.2023.1148781 PMC10235462

[28] Nosarti, C. et al. Grey and white matter distribution in very preterm adolescents mediates neurodevelopmental outcome. *Brain* **131**, 205–217 (2008). 18056158 10.1093/brain/awm282

[29] Strand-Brodd, K. et al. Development of smooth pursuit eye movements in very preterm infants: 1. general aspects. *Acta Paediatr.* **100**, 983–991 (2011). 21332783 10.1111/j.1651-2227.2011.02218.x PMC3123744

[30] Mori, S. et al. *MRI Atlas of Human White Matter* 1st edn (Elsevier, Amsterdam, the Netherlands, 2005).

[31] Makris, N. et al. Decreased volume of left and total anterior insular lobule in schizophrenia. *Schizophr. Res.* **83**, 155–171 (2006). 16448806 10.1016/j.schres.2005.11.020

[32] Frazier, J. A. et al. Structural brain magnetic resonance imaging of limbic and thalamic volumes in pediatric bipolar disorder. *Am. J. Psychiatry* **162**, 1256–1265 (2005). 15994707 10.1176/appi.ajp.162.7.1256

[33] Vangberg, T. R. et al. Changes in white matter diffusion anisotropy in adolescents born prematurely. *NeuroImage* **32**, 1538–1548 (2006). 16843682 10.1016/j.neuroimage.2006.04.230

[34] Sripada, K. et al. Visual-motor deficits relate to altered gray and white matter in young adults born preterm with very low birth weight. *NeuroImage* **109**, 493–504 (2015). 25592994 10.1016/j.neuroimage.2015.01.019

[35] Kochukhova, O. et al. Antenatal steroids and neurodevelopment in 12-year-old children born extremely preterm. *Acta Paediatr.* **111**, 314–322 (2022). 34617304 10.1111/apa.16140

[36] Kaul, Y. F. et al. MRI findings, looking behaviour and affect recognition in very preterm children: a pilot study. *Physiol. Behav.* **280**, 114553 (2024). 38615730 10.1016/j.physbeh.2024.114553

[37] Morita, T., Asada, M. & Naito, E. Contribution of neuroimaging studies to understanding development of human cognitive brain functions. *Front. Hum. Neurosci.* **10**, 464 (2016). 27695409 10.3389/fnhum.2016.00464 PMC5023663

[38] Hollund, I. M. H. et al. White matter alterations and their associations with motor function in young adults born preterm with very low birth weight. *NeuroImage Clin.* **17**, 241–250 (2018). 29159041 10.1016/j.nicl.2017.10.006 PMC5683190

[39] Lenroot, R. K. & Giedd, J. N. Brain development in children and adolescents: insights from anatomical magnetic resonance imaging. *Neurosci. Biobehav Rev.* **30**, 718–729 (2006). 16887188 10.1016/j.neubiorev.2006.06.001

